# The psychological clang of terrorism fear and stress on employees’ well-being

**DOI:** 10.3389/fpsyg.2025.1499371

**Published:** 2025-10-17

**Authors:** Syed Asad Ali Shah, Asmaa Alyaemni, Liaqat Ali

**Affiliations:** ^1^Department of Human Resources Management, College of Business, Alasala University, Dammam, Saudi Arabia; ^2^Department of Health Administration, King Saud University, Riyadh, Saudi Arabia

**Keywords:** job satisfaction, terrorism-induced stress, fear of terror, education sector, employees’ well-being

## Abstract

The education sector is a conspicuous target for terrorist attacks, significantly impacting the quality of life and well-being of its employees. Job satisfaction is a key indicator of employee well-being; thus, understanding the link between the fear of terror and job satisfaction is essential but remains unexplored. This study examines the association through the lens of terrorism-induced stress in the education sector of the two most terrorism-affected regions in Pakistan. Data were collected using a structural questionnaire and were analyzed through structural equation modeling techniques. This research study explored a significant relationship between the fear of terrorism and job satisfaction, which is partially mediated by terrorism-induced stress. Implications for improving a healthy work environment in terrorism-affected regions and recommendations for future studies based on the findings are discussed.

## 1 Introduction

Freedom from hunger and fear are the two integral elements that regulate human security (Wheeler, 2011). In line with Maslow’s hierarchy of needs, once basic physiological needs such as food and financial stability are met, individuals then seek safety and security, which are crucial to their overall well-being. While employers may meet their employees’ financial needs to satisfy their hunger, they cannot assure their workers’ freedom from terrorism, which is unpredictable and beyond their control. Terrorism is considered the intentional use of violence or threats to instill fear within the population to gain a particular political aim (Stankov, 2018). In today’s modern era, terrorism and extremism are considered a fact of the time and have recently appeared as an eminent social issue. Terrorist attacks are sudden, rare, uncertain, and vicious, which occur everywhere, but the Middle East, South Asia, and Africa are largely devastated regions (Tin et al., 2022; Gaibulloev and Sandler, 2019; Kim and Sandler, 2020). Terrorist attacks such as 9/11, Army Public School Peshawar 2014, Paris terror attack in 2015, Charsadda University attack in 2016, Christchurch mosque New Zealand attack in 2019, Kabul school bombing in 2021, Zamfara massacres Nigeria in 2022, Peshawar mosque bombing in 2023 and Crocus city hall attack Krasnogorsk, Russia in 2024 are the common examples. These incidents demonstrate that terrorist attacks are not confined to well-known hotspots but can occur everywhere, at any time, without any warning, and they can target anybody (Cohen-Louck, 2016; Vogel, 2024).

The South Asian Terrorism Portal reported that Pakistan has lost around 71,808 lives since 2001 (South Asia Terrorism Portal (SATP), 2025), with Balochistan and Khyber Pakhtunkhwa amongst the most affected regions. Human Right Watch (2017), revealed that frequent terrorist attacks in Pakistan targeted the education sector. From 2007 to 2022, the region of Khyber Pakhtunkhwa witnessed about 1,000 terrorist attacks on schools [South Asia Terrorism Portal (SATP), 2025]. Similar violence has been reported on the educational institutions of Balocihistan, disrupting schools, colleges and government offices (Human Right Watch, 2017). The major reason for such attacks is the ease of access to educational institutions (universities, colleges, and schools) due to inadequate measures of security compared to other sectors, making them more vulnerable (Malik et al., 2017). Moreover, the gathering of locals for co-curricular activities, like concerts, political events, and sports, could be the cause. While the disturbance of these events grabbed huge attention of the mass media. These terrorist attacks on the education sector in the near past, especially in South and South East Asian and Middle Eastern, African countries, have developed a global challenge (Tin et al., 2022; Kim and Sandler, 2020; Naja et al., 2016). Such attacks are more effective in getting an inevitable sense of helplessness and fear and are more powerful and persistent than any other disasters (Ryan et al., 2003; Elmas, 2021).

Educational sector employees were targeted by terrorists and faced a tenacious fear of attacks, and stayed with them for a long time. This sharp fear can lead to employees leaving their institutions for the sake of their life protection (Bader et al., 2016; Memon et al., 2022). This unpredictable situation leads to crucial stress and deteriorated attitudes toward one’s job, which eventually disturbs their quality of life (Ryan et al., 2003; Farooq Malik et al., 2014; Bader and Berg, 2013a; Raja et al., 2020). In addition, it has been revealed that unveiling terrorism has pernicious ramifications on employees’ psychological well-being, attitudes, and quality of life (Farooq Malik et al., 2014; Hurley-Hanson et al., 2011; Soomro et al., 2023) and detrimentally affects employee performance and efficiency (Boscarino et al., 2006; Rahman et al., 2020; Saleem and Malik, 2023). Based on the mentioned reasons and arguments, it is critical for all organizations, particularly educational institutions, to consider the negative effects of the external threat of terrorism and its impacts on their employees’ job satisfaction. By doing so, they can implement measures to mitigate these negative impacts. The literature suggests that job satisfaction in the service sector can be enhanced through job enrichment (Herzberg, 2003; Marta et al., 2021). Since in the terrorism affected areas job satisfaction cannot be achieved merely through higher pay or additional benefits. Instead, employees can find satisfaction through non-financial means, such as a secure and psychologically supportive work environment which can lead to an improved standard of comfort by reducing the adverse social marker associated with fear of terrorism (Hall, 2024).

To the best of our understanding and literature, no prior studies have specifically explored the link between fear of terrorism and job satisfaction in the education sector. Moreover, no research has empirically examined whether terrorism-induced stress mediates the relationship between fear of terrorism and employee job satisfaction. Existing studies in this area have predominantly focused on industrial employees and expatriates (Bader and Berg, 2013a; Glenn Richey and Reade, 2009; Owens and Johnson, 2024). This study addresses this gap under the domain of education department personnel in Pakistan, investigating the consequences of fear of terrorism on their job satisfaction, with a particular emphasis on terrorism-induced stress as a mediator. The proposed conceptual model has filled these gaps in the literature, by drawing on the Conservation of Resources (COR) theory proposed by Hobfoll (1989). This theory suggests that individuals fight to obtain, maintain, and protect their most valued resources. However, stress arises when there is a loss or threat of loss to these resources. In the given model fear of terrorism signifies one’s worries about losing their valuable resources, for example, their health and life, as well as the well-being of their family and friends. This fear and worry of losing valuable resources elevates stress levels, in individuals and leads to job dissatisfaction, anxiety, and quitting thoughts, ultimately producing a loss or strain on psychological and physical resources (Liu et al., 2008; Shah et al., 2020) and lowering their well-being in the catastrophic condition of terrorism. Based on COR theory, this study proposes that non-work-related terrorism-induced stress can spill over into the workplace and adversely affect employees’ job satisfaction.

### 1.1 Theoretical background and hypothesis

#### 1.1.1 Fear of terror and job satisfaction

Everyone is surrounded by various potential stressors, and an increasingly common stressor is terrorism, which is leading to stress and strain in general masses. Terrorist attacks create a sense of vulnerability and fear among the people, which is more invasive and persistent as compared to other types of catastrophes (Ryan et al., 2003; Soomro et al., 2023) and results in higher civilian casualties. These fears generated from terrorist attacks create a ripple in society and produce a threat perception that impacts numerous personal and work-related outcomes including job attitudes (Farooq Malik et al., 2014; Raja et al., 2020; Soomro et al., 2023; Shah et al., 2018). Attitudes are “the psychological tendency which is expressed after evaluating a particular entity with some degree of favor or disfavor” (Eagly and Chaiken, 2007). Job satisfaction is considered one of the most focal constructs of employees’ attitudes (Saari and Judge, 2004; Kauppila, 2024), well-being, and quality of life (Wang and Jing, 2017; Sirgy et al., 2018). Locke (1976), defined job satisfaction as “a pleasurable or positive emotional state resulting from the appraisal of one’s job or job experiences” (p. 1304). Based on this conceptualization, Hulin and Judge (2003) posited that job satisfaction represents a multifaceted psychological reaction to an individual’s job and environment. Working in a terrorism-endanger environment elicits the recognition of future terrorist incidents which in connection not only adversely affects the psychological functioning of their inhabitants but also affects their daily life routines (Shah et al., 2018), job attitudes, and quality of life (Malik et al., 2017). The security situation in terrorism-affected areas of Pakistan has deteriorated, and educational institutions are continuously under threat, it is a common sight to see the employees of this sector going through various security checks on the way to their work and thorough body searches at the entrance of their institutions as well. However, such measures are indispensable to ensure security, but these checks unconsciously remind them of the continuously existing threat of terrorism. Such continuous daily reminder affects their cognitive and affective state of mind and has a potential impact on their job satisfaction.

Consistent with the COR theory, we propose that when employees confront terrorism and its related stressful situations daily, it depletes their cognitive and psychological resources (Hobfoll, 1989; Hobfoll and Shirom, 2001). Such depletion in cognitive and psychological resources could lead to lower job satisfaction (Lee and Ashforth, 1996). Previous research studies have also shown that terrorist attacks have detrimental effects on employees’ work attitudes (Ryan et al., 2003; Farooq Malik et al., 2014; Glenn Richey and Reade, 2009; Reade and Lee, 2012). Ryan et al. (2003) examined the impact of the 9/11 terrorist attack on worker attitudes in the context of multinational companies within the U. S., reporting minimal effects on employees’ work attitudes and no significant changes in job satisfaction or stress levels. Similarly, Hurley-Hanson et al. (2011) in his study found that respondents mainly disagreed that job satisfaction was adversely affected by 9/11, following the attacks. Though, we are of the view that these results may be influenced by the rarity of terrorist incidents on U. S. soil, and focus on a single event. In contrast, the current study examines a region that has faced ongoing and frequent terrorist attacks, where constant exposure may have a deeper and more sustained impact on employees’ job satisfaction and overall well-being. Glenn Richey and Reade (2009) found that terrorism sensitivity is negatively correlated with organizational commitment and satisfaction. Moreover, the stressful situation created by the fear of potential terrorist attacks often leads to a state of burnout hence emotional and mental fatigue are common consequences of facing such situations daily (Shah et al., 2020; Shah et al., 2018; Soomro et al., 2021). Henceforth we argue that many employees of the education sector may develop negative attitudes toward their job since it requires them to work in a highly stressful environment, which may eventually lead to dissatisfaction from one’s job. Therefore, we proposed that:

*H1*: Fear of terrorism is negatively associated with employees’ job satisfaction.

#### 1.1.2 The mediating role of terrorism induced stress in the relationship between fear of terror and job satisfaction

Fear is often regarded as a profound and unsettling emotion that surfaces when one senses a looming threat or danger to oneself or society at large. The increase in global terrorism in recent years has raised the fear of becoming the victim of terrorist attacks (Kaakinen et al., 2021; Onat et al., 2024; Shechory-Bitton and Cohen-Louck, 2016; May et al., 2011). People in Pakistan are continuously under the threat of terrorism (Malik et al., 2021; Malik et al., 2020). What adds to their fear is the uncertainty about when and where such attacks may happen (Shah et al., 2020). Such uncontrollability of external threats leads to active reactions of the fear of terror, anxiety, and helplessness (Nasier, 2023; Gökyar and Erdur-Baker, 2022; Abiola et al., 2017). Moreover, educational sector employees are more vulnerable to such attacks (Naja et al., 2016; Rahman et al., 2020; Saleem and Malik, 2023). In the past, they have witnessed deadly incidents of terrorist attacks that occur randomly at various educational institutions, hence creating a high level of uncertainty for them (Naja et al., 2016; Rahman et al., 2020). It is believed that when employees are uncertain about their external environment, they feel constant fear, which in response increases their level of stress (Kaakinen et al., 2021; Onat et al., 2024; Nasier, 2023). In the context of education Memon et al. (2021) revealed that perceived threats lead to heightened job risk and job anxiety. Similarly, Saleem and Malik (2023) noted that exposure to terrorism positively impacts Post Traumatic Stress Disorder among university teachers. Previous studies have also shown that even those people who are peripherally influenced by terrorist attacks, sense imminent threat and personal lack of security and show a variety of symptoms of depression, anxiety, stress, trauma, and aberration (Chipman et al., 2011; Canetti et al., 2013; Yetzer and Pyszczynski, 2019; Freh and Cheung Chung, 2021).

Bader and Berg (2013a) in the context of expatriates, explored that terrorism and its related situational and role stressors and stress adversely affect employees’ attitudes and performance, which leads to dissatisfaction toward host country nationals and reduced work effectiveness. Conversely, we are of the view that education sector employees working in terrorism-endangered areas are in a constant state of fear and stress; similar attacks in the past have changed their perception that “this will not happen to me” is replaced by “when will this happen to me” (Cohen-Louck, 2016). This change in perception elicits an increasing vulnerability to the fear of terrorist attacks. The resultant high level of vulnerability along with the uncertainty about one’s external environment, leads to a substantial level of stress (Raja et al., 2020; Bader and Berg, 2013b). These arguments are second the COR theory that states that perceptions of sinking one’s valuable resources (i.e., individual life himself, their health and their peers and loved ones), elicit individual stress levels (Hobfoll et al., 2006). A study conducted by Kurniawaty et al. (2019) shows that working in a stressful environment leads to low job satisfaction and increase turnover intention. Furthermore, the impacts of stress on job satisfaction are extensively focused by various researchers showing the adverse effects of stress (Kim et al., 2024; Jaysan et al., 2024; Woods et al., 2023; Manzoor et al., 2011).

However, little attention has been paid to how terrorism-induced stress impacts employees’ job satisfaction. In an attempt to establish this potentially intuitive relationship, we propose that the pressure of working in an uncertain work environment (characterized by fear of terror) increases the level of stress and in turn, affects the cognitive, affective state of mind and general well-being which eventually results in decreasing the level of job satisfaction. These arguments align with Bader and Berg (2013b), who proposed that an increased level of stress from terrorism can influence employee performance and work attitudes, including job satisfaction. Similar reactions to stress employees’ decline in work concentration, and disconnectedness with organizations have been reported after the 9/11 terrorist attacks (Mainiero and Gibson, 2003; Cornwell et al., 2023; Nielsen et al., 2023). More specifically the study of Memon et al. (2022) explored that terrorism heightened job stress in teachers further leads to turnover intention. Raja et al. (2020) and Folkman (2013) argue that a high level of stress is supposed to have negative impacts on a variety of outcomes and job satisfaction is one of them. Even though the studies of Ryan et al. (2003) and Bader and Berg (2013a) indicated that terrorism induces perceived stress could mediate the relationship between terrorism sensitivity and employees’ work attitudes. However, so far none of the research has looked at the potential mediating role of terrorism-induced stress in the association between employees’ job satisfaction and their fear of terror.

To address this gap, based on the existing literature and the logical arguments, we propose that fear of terrorism may result in increasing the level of stress, and such terrorism-induced stress may have a negative impact on employees’ job satisfaction. Therefore, we propose the following:

*H2*: Fear of terrorism is positively associated with terrorism-induced stress.

*H3*: Terrorism-induced stress is negatively associated to employees’ job satisfaction.

*H4*: Terrorism-induced stress partially mediates the connection between the fear of terrorism and job satisfaction.

## 2 Methodology

### 2.1 Research design and sample

The current study population consists of the educational sector (institutions involved in learning, teaching, and academic administration) employees of Pakistan who were working in different cities of Baluchistan and KP. These regions have been chosen for investigation because of their contextual relevance and geopolitical importance. They have historically been experiencing a high number of terrorist attacks, making them particularly important for investigation. Additionally, both regions shared their boundaries with Afghanistan, a region historically been linked to militant activity and cross-border insurgency, since 9/11 (Shahbaz et al., 2013). Convenient-sampling techniques were used due to accessibility, cost, time and limitations. In line with the nature of our research and the challenges associated with reaching a broader, randomly selected sample, convenience sampling allowed us to gather relevant data efficiently while ensuring the study’s feasibility. The data for this study were collected from 46 schools, 25 colleges, and 17 universities employees who work in this sector such as teachers, deans, academics, and all other education personnel, including transport and support staff, from janitors to bus drivers and education officials. The participants were either approached via online or physical means by distributing a self-administered structured questionnaire. A brief introduction to the study was provided. It was conveyed that participation would be voluntary and their responses would be analyzed as a general without reference to their identities. A total of 447 respondents participated in this study, out of which 34 questionnaires were considered not relevant due to some incomplete/missing information, and 413 duly filled questionnaires were kept for further analysis. Out of 413 responses, 58.6% (*n =* 242) were male and 41.4% (*n =* 171) were female. Regarding age 16.2% (*n =* 67) were in the age group of 20–30 years, 40% (*n =* 165) were age in the age group of 31–40 years, 26.2% (*n =* 108) were between 41–50 years, and 17.7% (*n =* 73) were aged 51 years and above. The experience of employees was noted as 1–5 years, 6–10 years, 11–15 years and 16-plus years. The average experience level of the participant was 5.08 years.

### 2.2 Measurement instrument

For the measurement of the focal construct, we asked respondent their level of agreement by utilizing a five-point Likert scale ranging from 1 (“strongly disagree”) to 5 (“strongly agree”). We used well-established and validated scales from previous studies. These scales were slightly modified according to our study context. To ensure measurement independence, including the rationale for each modification, wording adjustments were made and reviewed through a panel of professors in the current research area, undertaken to ensure content validity. Moreover, a pilot study was conducted to further review and refine survey items. Furthermore, post-data collection, exploratory factor analysis (EFA) for each construct of the study was performed, which confirmed that all the item scales were loaded on their respective construct. Moreover, the construct validity was measured and verified that all the scale items were empirically distinct constructs in the current study. The items for measuring fear of terror were adopted from terrorism catastrophizing which was developed by Sinclair and LoCicero (2007). This scale consists of 13 items with a composite reliability of 0.961 in the given study, sample items from the scale are “I have difficulty keeping the threat of terrorism out of my mind” and “There is little I can do to protect myself from terrorism.” Terrorism-induced stress was measured through a 4-item adapted scale from Cohen et al. (1983) which is the most widely used scale for measuring stress. The composite reliability of this scale was 0.875 in our study, and the sample items are “I feel that I am unable to control important things in my life because of the fear of terror” and “I feel that terrorism sensitivity affects my psychological ability.” Job satisfaction of employees was measured by a 5-item adapted scale of Brayfield and Rothe (1951). The overall reliability of this scale in our study was 0.891 and sample items from the given scale are “I feel fairly satisfied with my present job despite terrorism threat” and “Most days I am enthusiastic about my work and do not think about terrorism and its related fear.” The composite reliabilities of all the scales are given in Table 1.

**Table 1:** Values of combined reliability, average variance extraction, maximum shared variance, and its square roots.

Variables	CR	AVE	MSV	Square roots of AVEs
Fear of terror	0.961	0.656	0.256	0.810
Terrorism induced stress	0.876	0.640	0.256	0.800
Job satisfaction	0.879	0.594	0.139	0.771

CR, AVE = Average variance extraction, Composite reliability, MSV, Maximum shared variance **p < 0.01.

### 2.3 Control variables

This study controlled three demographic variables that are age, gender, and experience. First, age (in years) because it is assumed that the ability to cope with stressful and challenging situations changes with one’s age (Froese and Peltokorpi, 2013). Second, this study controls for the gender (1 = male, 2 = female), because previous studies show that gender might influence the fear or perception of terrorism. The study of Reade and Lee (2012) indicates that females are more sensitive to ethnopolitical conflict and have lower job satisfaction in those countries and areas where the terrorism level is high (Bader et al., 2015). Third, this study controlled for experience (in years) because experienced employees are better able to cope with stressful situations (Froese and Peltokorpi, 2013).

## 3 Data analysis and results

SPSS version 23.00 was used for the initial analysis of data. Descriptive statistics mean, standard deviation and correlation were calculated for the study variables (see Table 2). As it is shown that older and more experienced employees indicated lower level of terrorism induced stress and higher job satisfaction. Moreover, gender differences influence stress perception and satisfaction. Furthermore, to determine the fineness of the model, and to test the hypothesis, the in-hand data were analyzed via confirmatory factor analysis (CFA) by utilizing the structural equation modeling (SEM) through AMOS 23. We further tested our scales for reliability, convergent validity, and discriminant validity as well.

**Table 2:** Descriptive analysis (standard deviations, means, and correlation among variables.)

Variables	Mean	SD	Age	Gender	Experience	FOT	TIS	JS
Age	2.45	0.96	1					
Gender	1.41	0.49	−0.150**	1				
Experience	5.08	4.20	0.386**	−0.112*	1			
FOT	3.51	1.23	−0.026	0.073	−0.186**	1		
TIS	2.92	0.95	−0.193**	0.153**	−0.271**	0.541**	1	
JS	2.69	1.13	0.218**	−0.196**	0.260**	−0.255**	−0.279**	-

SD, Standard deviations. **P < 0.01. *p < 0.05. FOT, Fear of terror; TIS, Terrorism induced stress; JS, Job satisfaction.

### 3.1 Model fit (validity and reliability)

CFA was conducted to assess whether the measurement model fit the data. Fit indices were computed to verify the SEM fitness with our data set. The result of the model fit indices exhibits that the data is well fit to the model. Further, explicitly, *χ*^2^ = 334.786, *χ*^2^/df = 1.625, df = 206, the comparative fit index (CFI) = 0.980, the Tucker-Lewis index (TLI) = 0.978, goodness of fit index (GFI) = 0.929, normed fit index (NFI) = 0.950, adjusted goodness of fit index (AGFI) = 0.912 and the root mean square error of approximation (RMSEA) was noted 0.039, all indicating a good model fit (Bagozzi and Yi, 2012; Marcoulides and Schumacker, 2013). Furthermore, the approximation of CFA shows the three factors model best fit the data. All the items were loaded well on their corresponding factors with factor loadings of grander than 0.50 thus showing that the items were good indicators of the given constructs. Table 1 shows the values of maximum shared variance (MSV), composite reliabilities (CR), square roots of AVEs and average variance extracted (AVE) of the study constructs. Values of all the CR were passed the range of the threshold of 0.7 showing a highly reliable. The AVE values were >0.5 for all constructs showing a high level of convergent validity, and the square root of AVE was noted as greater than the inter-construct correlations for all the variables showing a high level of discriminant validity (Murtagh and Heck, 2012; Fornell and Larcker, 1981). To further prove the discriminate validity, the researcher specifies that it can be achieved when the MSVs values are less than the values of AVEs. The results showed that MSVs for all the constructs were less than AVEs thus further establishing discriminant validity. Furthermore, as we mentioned earlier for the testing of our study hypotheses we used SEM by using AOMS, which exhibits a good fit with the data (*χ*^2^ = 402.218, df = 263, *χ*^2^/df = 1.529, CFI = 0.979, GFI = 0.926, TLI = 0.976, NFI = 0.942, AGFI = 0.908, and RMSEA = 0.036).

### 3.2 Hypothesis testing

#### 3.2.1 The direct relationships among study variables

**Figure 1:**
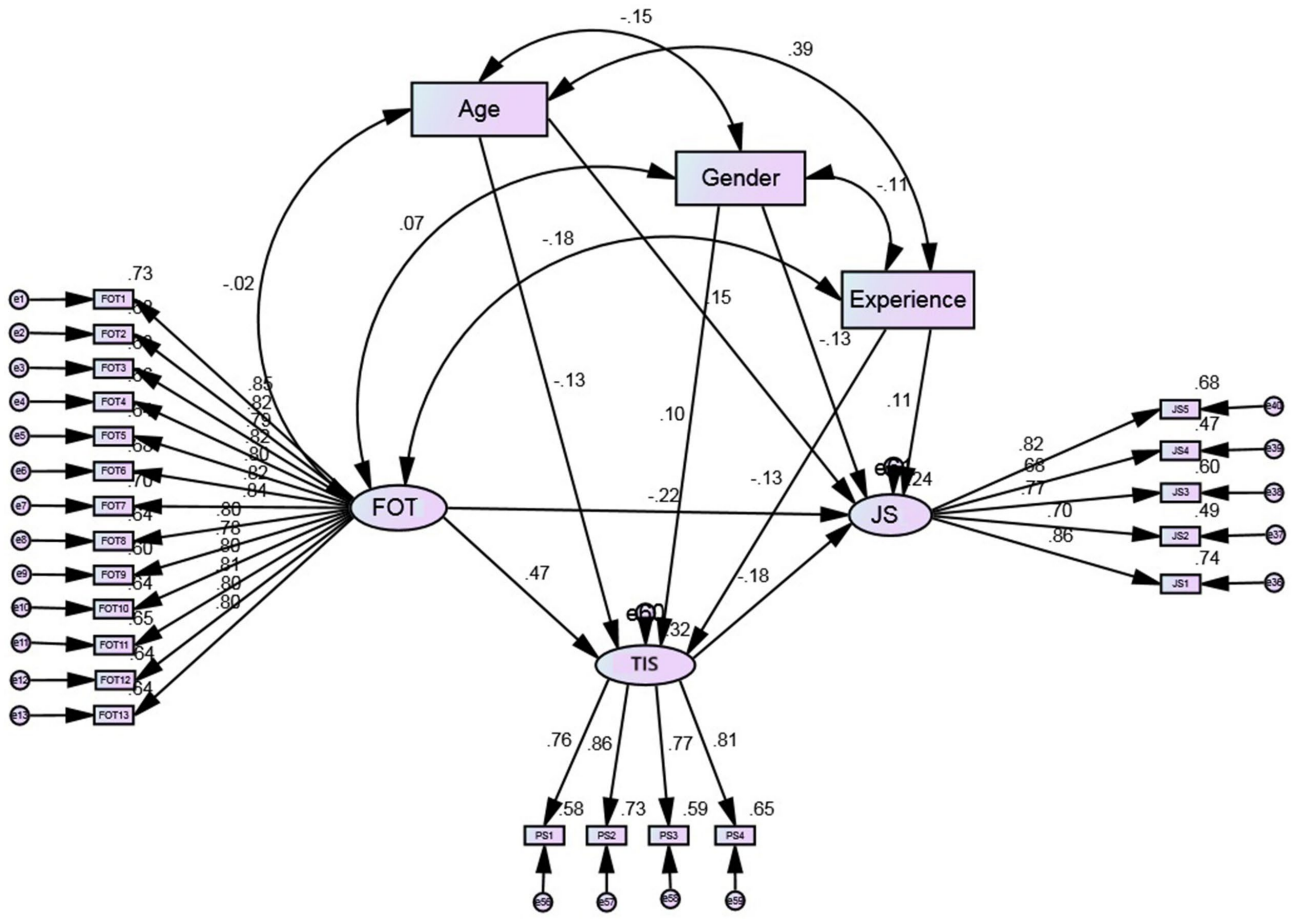
SEM model.

The Figure 1 (structural model) shows the relationship among study variables. The results show a significant negative association between job satisfaction and the fear of terror (*β* = −0.22, C. R = −3.804, *p <* 0.01). Hence, supporting H1. The direct path from fear of terror to terrorism-induced stress was positive and significant (*β* = 0.47, C. R = 9.028, *p <* 0.01) supporting H2. The path from terrorism-induced stress to job satisfaction was negative and significant (*β* = −0.18, C. R = −2.855, *p <* 0.01). Hence, H3 was also supported.

#### 3.2.2 Indirect mediating effect of terrorism induced stress

The indirect effects of fear of terror on employees’ job satisfaction through terrorism-induced stress were analyzed with the help of bootstrapping in AMOS. The bias-corrected bootstrap confidence interval was calculated which is considered as one of the best methods for testing and analyzing the mediation effects (Cheung and Lau, 2008). We used 95% biased corrected confidence intervals with 5,000 bootstrap samples to check the indirect effect of the fear of terror on job satisfaction mediated by terrorism-induced stress.

The lower and upper bound for the indirect effect interval do not contain zero which supports the significance of the hypothesis as per conventional standards. The results in Table 3 show that the indirect effects of fear of terror on job satisfaction (*β* = −0.085, *p <* 0.01, 95% CI = [−0.154, −0.021]) were significant thus indicating that terrorism-related stress significantly intervened the connection between terrorism fear and job satisfaction. Hence, H4 was supported as well.

**Table 3:** The indirect mediated effect of terrorism-induced stress between job satisfaction and terrorism fear.

Variable	Standardize indirect effects (*β*)	95% biased corrected confidence interval	
		Lower bounds	Upper bounds
Fear of Terror	−0.085**	−0.154	−0.021

The bootstrap sample numbers = 5,000. **P < 0.

## 4 Discussion

Previous studies confirmed detrimental impacts of the fear of terrorism and exposure on employees’ mental health, life satisfaction, and job attitudes (Farooq Malik et al., 2014; Raja et al., 2020; Soomro et al., 2023; Shah et al., 2020; Shah et al., 2018; Farzanegan et al., 2017). However, the effect of terrorism fear on employee job satisfaction has been ignored by the previous literature. This article discussed how terrorism fears cause stress and strain, and as a result, it impedes employee attitude of job satisfaction. This study based arguments on the COR theory and suggested that a non-work related stressor, i.e., fear of terror can penetrate the workplace and impact employees’ job satisfaction. These findings are consistent with the previous studies of Glenn Richey and Reade (2009), Bader et al. (2020), and Bader and Berg (2013a) which indicated that terrorism sensitivity affects employees’ job attitudes specifically satisfaction with their jobs. In the same way, Ryan et al. (2003) and Hurley-Hanson et al. (2011) explored the positive relationship between the 9/11 terrorist attacks and employees’ attitudes. Though our study revealed a significant negative relationship between the fear of terror and employees’ job satisfaction. These differences in results may be influenced by the rarity of terrorist incidents in the United States of America and the frequent terrorist attacks in Pakistan. As discussed earlier, in the regions of Baluchistan and KP provinces of Pakistan, the terrorists had frequently targeted the education sector. Hence, the employees of this sector are under a constant fear of terror that strains their cognitive and affective psychological resources, and thus employees feel less satisfied with their jobs and life since they consider the job as a source of exposure to such a threatening environment. These findings are consistent with the conservation of resource theory, which states that when employees face such stressful situations (terrorism-related), they lose their valuable psychological resources (Hobfoll et al., 2018). Such loss of resources may result in lower job satisfaction when the cause of the loss of resources is associated with the workplace and the nature of the job (Lee and Ashforth, 1996).

The second hypothesis H2, terrorism fear has a direct positive effect on terrorism-related stress was also supported. The findings align with an earlier study by Bader and Berg (2013a) and Sarwar et al. (2020) which illustrated that terrorist threats and their related activity could increase the level of stress in individuals. When employees are fearful about terrorist attacks, they frequently think that they or their loved ones might be the next victim of it. Hence, it increases their level of stress due to terrorism. Such terrorism-induced stress is seen to be common in the terror-affected areas of KP and Baluchistan. The third hypothesis H3, stating that terrorism-induced stress is negatively related to job satisfaction was also supported, indicating that when employees are in stressful conditions mainly due to their unsafe workplace environment, their dissatisfaction with the job increases. Studies have shown that when the fearful situation due to terrorism threat increases employees’ stress, they concentrate more on the safety of themselves and their family members rather than on the job (Kastenmüller et al., 2011). Similar arguments are provided by the assumptions of terror management theory, which states that when people face an uncertain situation, they neglect their work and concentrate more on their families (Raja et al., 2020; Kristensen, 1991). These arguments by previous theories and studies are consistent with the current study results. Moreover, the results of the current study are also in line with the previous findings of Pinelli et al. (2024) and Watson et al. (2010) stating that perceived stress can cause employees’ job satisfaction. The fourth hypothesis H4, depicting that terrorism-induced stress works as a mediating mechanism between fear or terror and the employee attitude toward job satisfaction was also supported. These results are in line with the prior studies of Bader and Berg (Bader and Berg, 2013a), Rubaltelli et al. (2018) argued that terrorism sensitivity increases the stress level of employees in terror-affected areas. Similarly, another study conducted by Itzhaki et al. (2018), showed that stressed employees at work are less satisfied with their jobs. COR theory states that for those individuals who try to obtain, maintain, and protect their valued resources, the fear of losing such resources increases their stress, which in response leads to physical or psychological strain (Hobfoll, 1989, 2002). In the context of the current study, the fear of terror represents individuals’ concern about losing their valuable resources of health and life, which increases the level of stress and hence leads to job dissatisfaction.

Summing up the discussion, this study has established a comprehensive understanding of the consequences of terrorism fear on workers’ well-being and job satisfaction. In addition, this study highlighted the previously unexplored mediating effect of terrorism-induced stress in the connection between terrorism fear and job satisfaction by indicating that terrorism-induced stress acts as a mechanism of transferring the effects of fear of terror on employees’ job satisfaction.

## 5 Theoretical and practical implications

Previous research showed that fear of terrorism is relatively unexplored in organizational settings. Only a few studies have focused on the impact of the fear of terrorism on employee’s attitudes (Ryan et al., 2003; Farooq Malik et al., 2014; Bader and Berg, 2013a; Hurley-Hanson et al., 2011). Considering the scarce literature on terrorism-related fear and employee attitudes, the given study has extended the current knowledge by exploring the effect of fear of terrorism on employees’ well-being, specifically job satisfaction. This study made another contribution to the literature by also exploring the mediating role of terrorism-induced stress. We founded our arguments on the conservation of resource theory Hobfoll (1989), and empirically illustrated how fear of terrorism influences employees’ job satisfaction through terrorism-induced stress.

These findings of the study provide some very important and real-world implications for managers or supervisors in terrorism-endangered areas. The mere recognition of the fact that terrorism threats reduce employees’ well-being and their job satisfaction should be enough to compel management to take practical steps to reduce, if not eliminate, such an adverse effect. This might be of great value for all organizations, specifically educational institutions whose employees are working under the constant threat of terrorism. To involve them in such activities, which are intended to reduce their fear of being the victim of terrorism and subsequently diminish their level of stress by providing a healthy work environment. These activities may include physical exercise and meditation, which are found to be very useful in reducing stress (Stoyva and Carlson, 1993). At the individual level, Acceptance and Commitment Therapy (ACT), Cognitive Behavioral Therapy (CBT), and Mindfulness-Based Stress Reduction (MBSR) have shown effectiveness in encouraging emotional regulation, resilience, and reducing unpleasant trauma symptoms (Mohammadzadeh et al., 2024). These therapies might be implemented on an individual level for coping with terrorism and its related stress and fear. At the organizational level, establishing networks among peers, fostering a psychologically safe working environment, conducting post-incident reflection sessions, and providing access to counseling might minimize the long-term effects of terrorism. Better security measures and clear safety communication can also help in lowering employees’ perceived stress and terrorism related fear. In addition, body-centered group interventions like Biodanza: movement-based therapy, integration of music and social interaction have shown substantial benefits in the reduction of stress and emotional regulation, offering potential recovery. These strategies will not only improve their well-being but will also facilitate employees in their workplace and enhance their job satisfaction. The study of Byron and Peterson (2002), reported that the employers of those organizations that provided supportive responses to their employees after the 9/11 attacks on the US in 2001 were more satisfied with their jobs. Furthermore, management should try to create a healthy work environment where employee’s resilience could be enhanced. The provision of training and psychological counseling to an employee may alleviate their stress level in promoting a healthy work environment.

## 6 Limitations and future research direction

This research has some limitations. First, we took samples for this study from two provinces of Pakistan, and these provinces are considered more vulnerable to terrorist attacks so we cannot generalize our result to the broader population who are not frequently exposed to such terror attacks. Second, collecting data via a convenient sampling technique assists access to the respondents, although it might limit the generalizability of the results and cannot amply for broader population representation. So the result may be carefully considered while applying it in an alternate context. Future scholars may use random or stratified sampling for broader implications to enhance the external validity. Third, we collected data through a single data collection source of structured questionnaires, so there might be the possibility of social desirability bias (Smith and Glass, 1987). However, the researcher tried to reduce such bias by ensuring the involvement of the participant was voluntary and anonymous.

Future studies need to conduct comparative evaluations for a better understanding of how terrorism and its impact could be perceived in different nations. Furthermore, research scholars might focus on other sectors: law enforcement employees, health workers, and expatriates. Employees of these sectors are more often targeted by terrorist attacks. Furthermore, future studies could consider examining the mediating role of job-related anxiety and the moderating effect of social support, self-efficacy, and mindfulness in the connection between fear of terror and job satisfaction for promoting a healthy work environment.

## 7 Conclusion

The research findings not only expanded the body of knowledge but also provided some significant practical insights related to the constructs under consideration. Summing up, it might not be possible for employers to eliminate the negative impacts of the fear of terror and its subsequent effects on the job satisfaction of employees, however, the mere acknowledgment of the existence of such effects has important practical implications. Hence, employers should seriously consider making all possible efforts to not only maintain a healthy and secure work environment but also provide psychological counseling to the affected employees so that the adverse effects of such fear are minimized.

## Data Availability

The raw data supporting the conclusions of this article will be made available by the authors, without undue reservation.
